# Leaf shrinkage: a predictive indicator of the potential variation of the surface area-to-volume ratio according to the leaf moisture content

**DOI:** 10.1186/s40064-016-2900-3

**Published:** 2016-08-02

**Authors:** Salaheddine Essaghi, M’hamed Hachmi, Mohammed Yessef, Mohammed Dehhaoui

**Affiliations:** 1 Institut Agronomique et Vétérinaire Hassan II, BP 6202, Rabat-Instituts, Rabat, Morocco; 2 Ecole Nationale Forestière d’Ingénieurs, BP 511, Tabriquet, 11015 Salé, Morocco

**Keywords:** Dimensional shrinkage, Leaves and needles, Foliar SVR

## Abstract

Leaf shrinkage provides insights into the potential variation of foliar SVR, within the same species, when leaf moisture content is changing in response to water deficit. Since SVR is among the most significant plant flammability features, leaf shrinkage would be a relevant component of fuel hazard assessment through its influence on SVR, enhancing—if it is taken into account—thereby the wildfire prediction accuracy. The purpose of this work is, first, to consider the leaf shrinkage by characterizing the plant species towards the shrinkability of their leaves, taking account the possible site effect, to characterize the behavior of shrinkage as a function of moisture content and finally to perform a classification for some dominant Mediterranean species based on the shrinkage levels. The assessment of the hierarchical relationships between the dimensional shrinkages is also aimed. Leaves and needles of thirteen tree and shrub species were harvested from six different sites in western Rif Mountains. Leaves dimensions and moisture content were measured regularly during a gradual drying at the laboratory. Dimensional shrinkages were calculated at each moisture content level. Dimensional shrinkages behaved similarly whether in leaf or timber and kept the same reporting relationships between each other. Among the species sampled in different sites, site effect is significant only in *Pinus canariensis* and *Pistacia lentiscus*. A classification of the plant species was carried out in three separate classes. Generally, shrinkage class of the plant species studied gave an idea on its flammability ranking reported in the literature, implying thus a cause-and-effect relationship between both parameters.

## Background

Fire risk assessment requires a realistic flammability ranking of the forest species (Dimitrakopoulos 2001), which is a relevant component of forest fire management (Dimitrakopoulos 2001; Valette 1990; Liodakis et al. 2008). Wildfires occurrence is governed by several parameters. Vegetation can either increase fire intensity or act as a heat sink and mitigate the fire size (Pellizzaro et al. 2007).

Surface area-to-volume ratio is the best descriptor of fuel particle size (Dimitrakopoulos and Panov 2001; Fernandes and Rego 1998; Hachmi et al. 2011a). Besides being widely used in most of fire behavior prediction systems, it is often introduced as a significant factor of plant species flammability (Fernandes and Rego 1998; Papió and Trabaud 1990; White and Zipperer 2010). Surface area-to-volume ratio (SVR) is also a critical plant property, considered as central to fuel characterization and fire risk assessment (Fernandes and Rego 1998; Hachmi et al. 2011a). SVR varies not only from a species to another—as reported in the literature—(Dimitrakopoulos and Panov 2001; Fernandes and Rego 1998; Hachmi et al. 2011a; Papió and Trabaud 1990; Hernando et al. 1995; Pereira et al. 1995) but within the same species, it is also influenced by the leaf moisture content especially foliar SVR, since leaf thickness [major element of SVR (Hachmi et al. 2011a)] is strongly correlated with moisture content (Búrquez 1987; Bacelar et al. 2004). Indeed, leaf thickness decreases when moisture content decreases (Búrquez 1987) and according to SVR computing formula (Hachmi et al. 2011a), SVR rises making the plant material even more flammable. Thus, leaf thickness variation could significantly affect the flammability of the species and therefore must be taken into account in plant flammability assessment in order to enhance the efficiency of the fuel hazard prediction models. Thickness variation range can be better approached from the “leaf shrinkage” concept by drawing upon the wood shrinkage concept that is among the most important wood properties. Thus, the more important the leaf shrinkage is, the higher the potential increase of SVR will be. No report has previously considered the leaf shrinkage parameter during the evaluation of the pyric properties of plant species. The importance of the leaf shrinkage is its contribution in SVR variation, so modifying one of the most significant pyric properties. Besides, given that leaf shrinkage depends on thickness variation as a response to water deficit, it can be a means of assessing water status of the plant in xeric conditions, since thicker leaves contain a greater volume of water (Bacelar et al. 2004). The assessment of this parameter would be relevant to fuel hazard rating (Alexander and Cruz 2012; Pausas et al. 2012).

Otherwise, leaf shrinkage is the combination of dimensional shrinkages following the three dimensions of the leaf (thickness, width and length). It would make sense, therefore, if we studied leaf shrinkage with regard to its three components. In addition, given that leaf shrinkage is highly influenced by the moisture content, this cause-and-effect relationship may be also affected by site effect since leaf moisture content is governed by the ecological factors (Papió and Trabaud 1990).

The aim of this work is, first, to characterize plant species by their leaf shrinkage potential and its dimensional ranges and then, to assess the possible effect of site on leaf shrinkage and to characterize the behavior of shrinkage as a function of moisture content. The next objective is to classify the forest fuels examined based on their potential leaf shrinkage.

## Methods

### Study sites

Six sites were located throughout northwestern Morocco (western Rif Mountains). Each site had experienced no fires for at least 3 years and contained a suite of canopy and understory species characterizing the respective ecosystems. All the study sites are properties managed by the Moroccan High Commission for Forests and gather forests of Larache, Ahl Srif and Souk L’Qolla (Larache province), Tanaqoub, Dardara (Chefchaouen province) and Bellota forest (Ouezzane province) (Table 1). Each site was chosen according to an altitudinal gradient starting at the cork oak forests (Atlantic coast) to pine forests of Chefchaouen and in a purpose to cover most tree and shrub species.

**Table 1:** Distribution of the harvested species among sampling sites

Sampling sites	Altitude (m)	Longitude N	Latitude W	Species harvested
Larache	25	35°13′45.9″	6°14′25.0″	*Q. suber*, *P. pinea*, *C. salviifolius*
Ahl Srif	142	35°00′18.1″	5°41′26.5″	*P. canariensis*, *C. monspeliensis*, *C. siliqua*, *C. crispus*, *P. lentiscus*, *Q. suber*
Souk L’Qolla	263	35°5′2.5″	5°34′19.5″	*P. pinaster*, *A. unedo*, *C. albidus*, *C. monspeliensis*, *C. siliqua*, *P. lentiscus*
Tanaqoub	615	35°7′2.7″	5°26′59.1″	*Q. suber*, *C. monspeliensis*
Dardara	406	35°7′50.0″	5°17′23.7″	*P. canariensis*, *A. unedo*, *P. lentiscus*, *Q. suber*
Bellota	128	34°56′5.0″	5°31′56.1″	*P. canariensis*, *C. monspeliensis*, *V. tinus*, *Q. coccifera*

### Species selection and sampling

Canopy and understory species were chosen based on their abundance in the ecosystems of Western Rif. The tree species studied were *Pinus pinea* (stone pine), *Pinus pinaster* (maritime pine), *Pinus canariensis* (Canary Island pine), *Ceratonia siliqua* (carob tree)*, Quercus suber* (cork oak) and *Quercus coccifera* (kermes oak). Shrub species were *Arbutus unedo* (strawberry tree), *Cistus albidus* (grey-leaved cistus), *Cistus crispus* (wrinkle-leaved rockrose), *Cistus monspeliensis* (narrow-leaved cistus), *Cistus salviifolius* (sage-leaved rockrose), *Pistacia lentiscus* (mastic tree) and *Viburnum tinus* (laurustinus) (Table 1).

The species were selected according to their availability at the site. The samples were harvested in August 2014. Because leaves are considered the most flammable parts of the plants (Dimitrakopoulos and Papaioannou 2001), only leaves and needles were collected. To eliminate the possible effect of age, six leaves or needles samples of different size and morphologies were selected from each species and at each sampling site. A total of 120 leaves and 30 needles was harvested from the sites, placed into sealed plastic bags and transported in a cooler with ice. Samples were monitored at the Laboratory of Wildfire Research, Ecole Nationale Forestière d’Ingénieurs located in Salé, Morocco.

### Samples physical characteristics and moisture content monitoring

Once in the laboratory, measurements concerning length, width, thickness and weight of the live fresh foliage samples were taken as Hachmi’s et al. method (2011a). To assess the shrinkage variability of the samples depending on their moisture content, sample dimensions were monitored during their progressive drying. Samples were therefore placed inside papers and pressed the first days of drying, in order to keep their initial shape and still adapted to size measurements even dry. Dimensions and weight measurements are repeated at regular intervals until the samples dried. During the air-drying period, the first days when the drying rate is high, the morphological and weight measurements are taken every 12 h. Later, as the air-drying rate decreases appreciably, the measurements are performed every 24 h. Once the samples are completely air-dried, and in an objective to reach, progressively, very low levels of humidity feigning dead foliage and litter moisture content, the samples were placed in the oven gradually at different ascending temperatures (30, 35, 40, 50 and 60 °C) during 24 h for each oven temperature. At each oven temperature, the same latter measurements (thickness, width, length and weight) are carried out. Weights obtained after oven-drying samples at 60 °C during 24 h are considered as the samples oven-dry weight that will establish a base of calculation of the samples moisture contents during all drying stages.

Moisture content of each sample was computed based on oven-dry weight (Behm et al. 2004) as follows:

$${\text{Moisture}}\;{\text{content}}\;\left( \% \right) = \frac{{{\text{fresh}}\;{\text{instant}}\;{\text{weight}} - {\text{ovendry}}\;{\text{weight}}}}{{{\text{ovendry}}\;{\text{weight}}}} \times 100$$

Leaf shrinkage corresponding to each size, also called dimensional shrinkage, was calculated per plant species, sampling site and moisture content level using the following formula (Hiziroglu 2007):

$${\text{R}}\,\left( \% \right) = \frac{{{\text{size}}\;{\text{difference}}}}{{{\text{initial}}\;{\text{size }}}} \times 100$$

Total leaf shrinkage was also calculated using the sum of the three dimensional shrinkages for each species.

All weights were measured at the laboratory with a Kern^®^ ALJ 120-4 balance with a maximum of 120 g and accuracy to 0.1 mg. Length, width and thickness measurements were carried out using a digital electronic caliper Powerfix^®^ 0–150 mm Z11155.

### Statistical analysis

Species data were analyzed for species and sampling site effects using one-way ANOVA test, at 95 % confidence level, in IBM Statistical Package for Social Sciences (SPSS). Pairwise comparisons of means were performed using Duncan’s test.

## Results and discussion

### Dimensional shrinkages

In response to water loss, leaves and needles shrunk. This shrinkage was globally more visible thicknesswise than in the other directions (Table 2). This result is in accordance with Bacelar et al. (2004), which attributed the leaf shrinkage primarily to the thickness decrease. The highest thickness shrinkage values were recorded at *P. pinea* (48.07 %), *P. pinaster* (45.23 %) and at one site (Dardara) with respect to *P. canariensis* (43.00 %) and *P. lentiscus* (45.60 %). Otherwise, the lowest values belonged to *C. salviifolius* (19.81 %) and *P. canariensis* at two sites (Ahl Srif and Bellota, 10.93 and 4.53 % respectively). In the thickness direction, leaves of most species exceeded 25 % except for *C. salviifolius* and *P. canariensis* at two sites (Ahl Srif and Bellota).

**Table 2:** Leaf shrinkage

Species	Site	Shrinkage thicknesswise (%)	Shrinkage widthwise (%)	Shrinkage lengthwise (%)	Total shrinkage (%)	SD	CV
*A. unedo*	Souk L’Qolla	28.49 ± 4.25	6.35 ± 1.01	6.30 ± 0.87	41.15	8.20	19.93
	Dardara	38.00 ± 2.84	5.84 ± 0.40	4.93 ± 0.52	48.77	6.88	14.12
	Average	33.68 ± 2.78	6.08 ± 0.49	5.55 ± 0.51	45.31	8.15	17.99
*C. siliqua*	Ahl Srif	35.43 ± 2.50	3.60 ± 0.33	5.43 ± 0.33	44.46	5.46	12.28
	Souk L’Qolla	39.38 ± 1.97	5.51 ± 1.60	4.33 ± 0.56	49.23	6.14	12.48
	Average	37.23 ± 1.67	4.47 ± 0.77	4.93 ± 0.34	46.63	6.01	12.90
*C. albidus*	Souk L’Qolla	34.99 ± 1.86	11.58 ± 1.25	8.69 ± 1.20	55.26	2.79	5.05
*C. crispus*	Ahl Srif	27.14 ± 3.53	6.53 ± 1.00	6.24 ± 1.95	39.92	5.16	12.93
*C. monspeliensis*	Ahl Srif	33.63 ± 5.37	14.85 ± 3.15	7.52 ± 0.49	56.01	18.46	32.96
	Souk L’Qolla	26.63 ± 6.35	20.07 ± 4.52	8.05 ± 1.12	54.74	27.32	49.91
	Tanaqoub	17.92 ± 3.42	12.35 ± 2.94	6.37 ± 0.89	36.64	12.34	33.67
	Bellota	34.16 ± 8.80	9.13 ± 1.76	7.77 ± 0.64	51.06	20.15	39.47
	Average	27.82 ± 3.14	14.32 ± 1.77	7.41 ± 0.41	49.55	20.50	41.37
*C. salviifolius*	Larache	19.81 ± 3.14	11.40 ± 2.05	9.62 ± 0.99	40.82	11.81	28.93
*P. canariensis*	Ahl Srif	10.93 ± 1.43	14.29 ± 4.15	0.93 ± 0.09	26.14	4.68	17.89
	Dardara	43.00 ± 2.82	20.67 ± 1.28	1.17 ± 0.20	64.83	5.39	8.31
	Bellota	4.53 ± 2.43	5.25 ± 2.98	1.81 ± 0.56	11.59	2.86	24.65
	Average	25.36 ± 5.56	15.22 ± 2.28	1.27 ± 0.18	41.85	24.97	59.67
*P. pinaster*	Souk L’Qolla	45.23 ± 1.26	27.63 ± 2.54	1.63 ± 0.32	74.49	8.02	10.77
*P. pinea*	Larache	48.07 ± 3.02	34.43 ± 3.51	3.39 ± 1.07	85.89	2.41	2.80
*P. lentiscus*	Ahl Srif	36.88 ± 3.56	6.50 ± 0.68	2.51 ± 0.29	45.88	7.38	16.09
	Souk L’Qolla	35.83 ± 1.17	6.10 ± 1.23	3.63 ± 0.71	45.56	6.06	13.30
	Dardara	45.60 ± 4.17	22.22 ± 3.42	4.66 ± 0.97	72.48	6.29	8.68
	Average	38.67 ± 1.92	10.28 ± 2.00	3.46 ± 0.41	52.41	13.47	25.70
*Q. coccifera*	Bellota	26.12 ± 3.14	4.71 ± 0.68	3.34 ± 1.20	34.17	8.30	24.30
*Q. suber*	Larache	22.89 ± 2.57	3.15 ± 0.55	2.04 ± 0.58	28.09	5.75	20.49
	Ahl Srif	30.27 ± 2.99	6.39 ± 0.82	2.63 ± 0.77	39.29	6.10	15.52
	Tanaqoub	27.61 ± 2.31	5.82 ± 1.91	2.59 ± 0.27	36.02	9.54	26.49
	Dardara	25.55 ± 5.04	6.03 ± 0.72	2.48 ± 0.17	34.07	11.69	34.32
	Average	26.63 ± 1.58	5.26 ± 0.59	2.43 ± 0.27	34.32	8.72	25.42
*V. tinus*	Bellota	34.93 ± 2.52	4.90 ± 0.54	4.77 ± 0.93	44.60	5.11	11.46

Measurements of the shrinkages thicknesswise, widthwise and lengthwise ± standard error. Total leaf shrinkage per species given for each site followed by standard deviation and coefficient of variation

Shrinkages widthwise and lengthwise were almost equal except for pine needles, leaves of *C. albidus*, *C. monspeliensis*, *P. lentiscus* and *Q. suber*, where shrinkage widthwise was greater than shrinkage lengthwise. In the widthwise, the leaves of *C. siliqua* (4.47 %), *V. tinus* (4.90 %) and oak species (~5 %) were the samples that retracted least, whilst generally pine needles shrunk most (15–34 %). This shrinkage was <15 % in most species except for *P. pinea*, *P. pinaster* and *P. canariensis* at one site (Dardara). In the lengthwise, leaf shrinkage of *P. canariensis* (~1 %) and *P. pinaster* (1.63 %) was the lowest, whereas it was more enhanced in *Cistus* species (6.24–9.62 %).

Total shrinkages of *Cistus* species were close and varied from 39.92 % (*Cistus crispus*) to 55.26 % (*Cistus albidus*). *Pinus pinea* needles had the highest total shrinkage values (85.89 %), while *Q.coccifera* and *Q. suber* indicated the lowest values (34.17 and 34.32 % respectively) (Table 2). In the thickness direction, needles of *P. pinea*, *P. pinaster* and *P. canariensis* (Dardara) were almost similar. *Pinus pinaster* and *P. canariensis* showed also close leaf shrinkage lengthwise (~1 %). Oak species leaves displayed almost the same values concerning total shrinkage (~34 %), shrinkage thicknesswise (~26 %), widthwise (~5 %) and lengthwise (~3 %).

As regards the species that were harvested in several sites (Table 1), one-way ANOVA, at 95 % confidence level, was performed to test the site effect on total leaf shrinkage. The test showed no significant effect for most of these species with p values varying from 0.128 (*A. unedo*) to 0.354 (*C. monspeliensis*) except for *P. canariensis* and *P. lentiscus*, for which *p* value = 0.000. The three sites where *P. canariensis* needles were sampled indicated all statistical differences. This is due to anatomical differences in *P. canariensis* needles, owing to the environmental conditions variability (Grill et al. 2004). Regarding the leaves of *P. lentiscus*, only one site (Dardara) was significantly different from the other sites (Ahl Srif and Souk L’Qolla), considered similar. According to Barazani et al. (2003), there is a high polymorphism in *P. lentiscus* leaves. This warrants the significant shrinkage differences observed between certain sites.

**Fig. 1:**
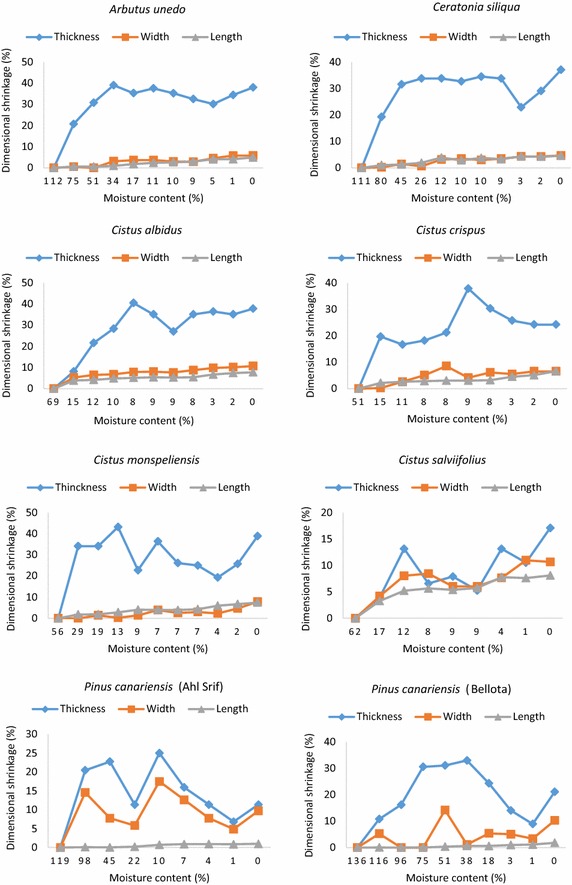
Dimensional shrinkages (%) as a function of leaf moisture content (%) taking into account the statistically different sites for *A. unedo*, *C. siliqua*, *C. albidus*, *C. crispus*, *C. monspeliensis*, *C. salviifolius *and *P. canariensis* (Ahl Srif and Bellota)

**Fig. 2:**
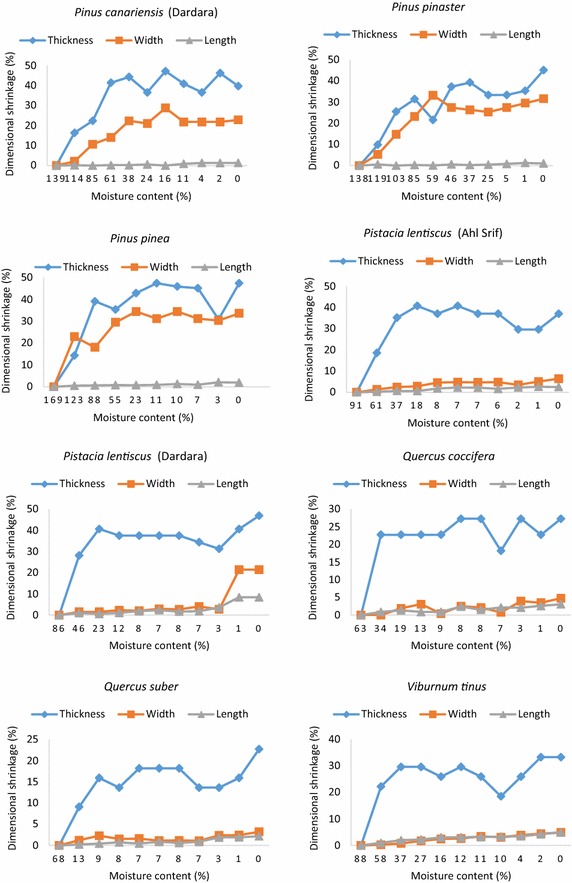
Dimensional shrinkages (%) as a function of leaf moisture content (%) taking into account the statistically different sites for *P. canariensis *(Dardara), *P. pinaster*, *P. pinea*, *P. lentiscus *(Ahl Srif and Dardara),* Q. coccifera*, *Q. suber *and *V. tinus*

 The evolution of leaf shrinkage in the direction of thickness, width and length (dimensional shrinkage) according to leaf moisture content is shown in Figs. 1, 2. Leaf shrinkages related to *P. canariensis* and *P. lentiscus*, which are significantly influenced by site effect, are presented in such a way as to cover all the statistically different sites. Leaf shrinkage in the thickness direction was by far the most dominant with regard to the shrinkages widthwise or lengthwise, whatever the species and the site (Figs. 1, 2). In the thickness direction, leaf shrinkage is even more important that the moisture content is low. In any case, shrinkage thicknesswise increased quickly at the beginning and then stabilized once a determined threshold is reached. Otherwise, the shrinkages lengthwise and widthwise generally merged and remained minors. Yet, the shrinkage widthwise got closer to the shrinkage thicknesswise for pine needles (all sites) (Figs. 1, 2), while in *C. salviifolius* leaves, the three shrinkages evolved jointly one beside the other, despite the persistent dominance of the shrinkage thicknesswise (Fig. 1).

The moisture content threshold below which the shrinkage thicknesswise stabilizes depended on the species, though some species had almost the same threshold. Indeed, all *Cistus* species displayed close thresholds (8–13 %). *Pinus pinaster* and *P. pinea* shrinkages both stabilized below the same threshold (83 and 85 % respectively). Species such as *A. unedo*, *Q. coccifera* and *V. tinus* showed close thresholds although they are not within the same genus. Pine needles had particularly the greatest thresholds (61–98 %), whilst *Cistus* species exhibited the lowest ones (8–13 %).

### Comparison with wood shrinkage

The anisotropic nature of timber leads to a shrinkage difference depending on the direction considered (Nepveu 1994). The tangential, radial and longitudinal anisotropy directions of timber correspond respectively to the thickness, width and length directions of the leaf. The shrinkage thicknesswise dominance over the others dimensional shrinkages is comparable to the dominance of the tangential shrinkage in wood over the radial and longitudinal shrinkages. Furthermore, the shrinkage widthwise is generally either greater or close to the shrinkage lengthwise, which is quite similar to the shrinkage behavior in wood. We conclude that the leaf behaves similarly to wood in response to drying.

### Forest fuels classification based on total leaf shrinkage

The comparison between total leaf shrinkage values related to the 13 forest fuels, through one-way ANOVA and Duncan’s multiple comparison test (95 % confidence level) showed highly significant statistical difference among the total foliar shrinkage values of the species examined (Table 3), which formed 3 levels of significance (Table 4). Most species fell into medium shrinkage category, whilst oak species were classified as low shrinkage species. *Pinus pinaster* and *P. pinea* showed greater leaf shrinkage values that enabled them to achieve the high shrinkage category (Table 4). Because of the significant site effect noted in *P. canariensis* and *P. lentiscus*, their leaf shrinkage rankings are therefore site-dependent and are not included in the classification table. *Pinus canariensis* was considered as less shrinkable species at Ahl Srif and Bellota (26.14 and 11.59 % respectively), but at Dardara, it can be regarded as moderately shrinkable (64.83 %). *P. lentiscus* leaves were classified as highly shrinkable (72.48 %) at Dardara. However, at Ahl Srif they were ranked as moderately shrinkable (45.88 %).

**Table 3:** Analysis of variance of total leaf shrinkage values of 13 forest fuels

Source of variation	SS	*df*	MS	*F* test	*F*
Method	16,596.204	12	1383.017	0.000 significant	7.004
Residual error	22,906.660	116	197.471		
Totals	39,502.864	128

**Table 4:** Some forest fuels classification according to their leaf total shrinkage

Shrinkage class	Species	Total shrinkage (%)
High shrinkage S > 65 %	*P. pinea*	85.89
	*P. pinaster*	74.49
Medium shrinkage 35 < S ≤ 65 %	*C. albidus*	55.26
	*C. monspeliensis*	49.55
	*C. siliqua*	46.63
	*A. unedo*	45.30
	*V. tinus*	44.60
	*C. salviifolius*	40.82
	*C. crispus*	39.92
Low shrinkage S ≤ 35 %	*Q. suber*	34.32
	*Q. coccifera*	34.17

### Leaf shrinkage versus drought-coping mechanisms and plant flammability

Leaf shrinkage, since it is primarily related to thickness variation, is determined by the amount of water inside the leaf (Bacelar et al. 2004; Bussotti et al. 2002) and the ability of the leaf cells to lose turgor after a water loss under drought conditions (Bussotti et al. 2002). Indeed, water stress induces an increase in cell wall elasticity (Martínez et al. 2007; Piñol et al. 1998) in preparation for leaf cells contraction (Martínez et al. 2007), resulting in leaf shrinkage. However, in order to cope with drought conditions, some adaptive mechanisms can mitigate this response (Aranda et al. 2014; Bacelar et al. 2004; Bussotti et al. 2002). Besides the stomatal regulation, sclerophylly is among the most common drought-coping plant feature (Aranda et al. 2014). Sclerophylly is enhanced by building parenchymatous tissues and the upper cuticular layer (Bacelar et al. 2004; Bussotti et al. 2002), increasing thereby the leaf tissue density (Aranda et al. 2014; Bussotti et al. 2002) and avoiding desiccation (Aranda et al. 2014; Pardos et al. 2009; Bacelar et al. 2004; Bussotti et al. 2002). The increased tissue density and water conservation both help to limit leaf shrinkage, since the parenchymatous tissues act as reinforcement support for the leaf internal components (Grill et al. 2004) and the water retention maintains the leaf turgor (Bacelar et al. 2004).

Indeed, the sclerophyllous character of the leaves of oak species made them least shrinkable as shown in the shrinkage classification table (Table 4). This feature enables the oak leaves to remain turgid as possible and therefore reduces the shrinkage potential. The leaves of *Cistus* species are less sclerophyllous than those of oaks, though they have a relatively thick cuticle that can mitigate the hydric stress effects (Aranda et al. 2014; Pardos et al. 2009; Bacelar et al. 2004; Bussotti et al. 2002). Therefore, *Cistus* leaves are classified as moderately shrinkable. The high shrinkage potential of *P. pinaster* and *P. pinea* can be explained by the prominence of two main parameters. Pine needles have, first, thin cuticle, allowing increased cuticular transpiration (Aranda et al. 2014; Pardos et al. 2009) and, secondly, pine needles are less sclerophyllous, depriving thereby the leaf of a mechanism limiting uncontrolled transpiration (Grill et al. 2004). This results in high desiccation susceptibility of the needles (Grill et al. 2004), which increases therefore the shrinkage potential (Bacelar et al. 2004). Otherwise, *P. canariensis* needles are less shrinkable than the other pine needles (Table 2). *Pinus canariensis* needles distance themselves from other pine needles through their particular epistomatal chamber, with a narrow aperture, where wax tubes are very dense and their small stomata compared to other pine species (Grill et al. 2004). These characteristics are responsible for the stomata occlusion (Jimenez et al. 2000; Zellnig et al. 2002). Thus, this arrangement of stomata cells inhibits transpiration (Zellnig et al. 2002) in order to limit water loss and then mitigate leaf shrinkage (Bacelar et al. 2004; Bussotti et al. 2002). In addition, cuticular transpiration in *P. canariensis* needles is the lowest among *Pinus* species (Pardos et al. 2009), showing even more fitness to keep water inside the needles, decreasing thereby the shrinkage potential (Bussotti et al. 2002). Furthermore, leaf shrinkage in *P. canariensis* varies significantly from a site to another. This result is in accordance with the following reports Climent et al. (2002), Grill et al. (2004), López et al. (2007) and Pardos et al. (2009) that highlighted a variation in the physiological response to hydric stress of *P. canariensis* needles from one site to the other. According to Grill et al. (2004), this is due to the variable anatomy of *P. canariensis* needles, which changes depending on environmental conditions.

Leaf shrinkage gives an idea on the potential of the leaf to change its dimensions and particularly its surface area-to-volume ratio when it dries and becomes litter. This potential is even more important to assess when we know that litter is the most vulnerable forest fuel towards wildfire. Indeed, litter is the forest fuel that has the greatest ignitability and combustibility (Liodakis et al. 2008). Afterward, the positive dimensional shrinkage values (Table 2) showed that all the dimensions studied decreased substantially—mainly thickness—as the drying and according to Hachmi’s et al. (2011a) SVR formula, SVR would have increased as a result. Species ranking in terms of their leaf shrinkage implies a ranking of the potential SVR variation range and subsequently help in wildfire prediction and fire hazard assessment (Fernandes and Rego 1998; Hachmi et al. 2011a; Hernando et al. 1995; Brown 1970; Rothermel 1983). In addition, since SVR is a characteristic favorable to flammability (Dimitrakopoulos and Panov 2001; Fernandes and Rego 1998; Hachmi et al. 2011b; Papió and Trabaud 1990; Pereira et al. 1995), the leaf shrinkage ranking of a species should be positively correlated with its corresponding flammability class. Our results showed that comparisons between shrinkage class of a plant species and its flammability class as reported in the literature revealed two different scenarios. Shrinkage classes of species such as *A. unedo*, *C. siliqua*, *C. albidus*, *C. crispus*, *C. monspeliensis*, *C. salviifolius* and *V. tinus* provided insights into their corresponding flammability classes as suggested by Dimitrakopoulos (2001), Dimitrakopoulos and Papaioannou (2001), Liodakis et al. (2008) and Hachmi et al. (2011b). Although the leaves of *Quercus* species were less shrinkable, they were regarded as flammable by the latter reports. This may be due to the numerous parameters involved in the determination of the species flammability, such as ash content, bulk density, fuel loading by size class and heat content (Dimitrakopoulos and Panov 2001; Papió and Trabaud 1990; White and Zipperer 2010; Behm et al. 2004). As leaf shrinkage in *P*. *canariensis* and *P*. *lentiscus* was site-dependent, their flammability would be also influenced by site effect and even related to other flammability parameters.

## Conclusion

This study focuses on leaf shrinkage—in the directions of thickness, width and length—in relation to moisture content; and the classification of forest fuels based on their leaf potential shrinkage. Future research prospects are conceivable for the extension of the shrinkage database in more plant species and for further clarification of the relation between leaf shrinkage and plant flammability. We conclude from this work that shrinkage, whether in timber or in leaf, behaves in the same way and the shrinkages in the three directions maintain the same reporting relationships between each other. Besides, leaf shrinkage raises when the moisture content decreases. *Pinus canariensis* needles are globally distinguished from other pine needles by low shrinkage and therefore show more drought-resistance ability, in accordance with the features of these needles as reported in the literature. Species of the genera *Cistus* and *Quercus* are respectively moderately and less shrinkable by dint of their mechanisms of drought tolerance.

## References

[CR1] Alexander ME, Cruz MG (2012). Assessing the effect of foliar moisture on the spread rate of crown fires. Int J Wildland Fire.

[CR2] Aranda I, Ramírez-Valiente JA, Rodríguez-Calcerrada J (2014). Características funcionales que influyen en la respuesta a la sequía de las especies del género *Quercus*: variación inter- e intra-específica. Ecosistemas.

[CR3] Bacelar EA, Correia CM, Moutinho-pereira JM, Gonçalves BC, Lopes JI, Torres-Pereira JMG (2004). Sclerophylly and leaf anatomical traits of five field-grown olive cultivars growing under drought conditions. Tree Physiol.

[CR4] Barazani O, Dudai N, Golan-Goldhirsh A (2003). Comparison of Mediterranean *Pistacia lentiscus* genotypes by random amplified polymorphic DNA, chemical, and morphological analyses. J Chem Ecol.

[CR5] Behm AL, Duryea ML, Long AJ, Zipperer WC (2004). Flammability of native understory species in pine flatwood and hardwood hammock ecosystems and implications for the wildland-urban interface. Int J Wildland Fire.

[CR6] Brown J (1970). Ratios of surface area to volume for common fine fuels. For Sci.

[CR7] Búrquez A (1987). Leaf thickness and water deficit in plants: a tool for field studies. J Exp Bot.

[CR8] Bussotti F, Bettini D, Grossoni P, Mansuino S, Nibbi R, Soda C, Tani C (2002). Structural and functional traits of *Quercus**ilex* in response to water availability. Environ Exp Bot.

[CR9] Climent J, Gil J, Pérez L, Pardos E (2002). Effecto de la procedencia en la supervivencia de plantulas de *Pinus canariensis* Sm. en medio arido. Investig Agrar Sist Recur.

[CR10] Dimitrakopoulos AP (2001). A statistical classification of Mediterranean species based on their flammability components. Int J Wildland Fire.

[CR11] Dimitrakopoulos AP, Panov PI (2001). Pyric properties of some dominant Mediterranean vegetation species. Int J Wildland Fire.

[CR12] Dimitrakopoulos AP, Papaioannou KK (2001). Flammability assessment of Mediterranean forest fuels. Fire Technol.

[CR13] Fernandes PM, Rego FC (1998). A new method to estimate fuel surface area-to-volume ratio using water immersion. Int J Wildland Fire.

[CR14] Grill D, Tausz M, Pöllinger U, Jiménez MS, Morales D (2004). Effects of drought on needle anatomy of *Pinus canariensis*. Flora.

[CR15] Hachmi M, Sesbou A, Benjelloun H, Bouanane F (2011). Alternative equations to estimate the surface-to-volume ratio of different forest fuel particles. Int J Wildland Fire.

[CR16] Hachmi M, Sesbou A, Benjelloun H, El Handouz N, Bouanane F (2011). A simple technique to estimate the flammability index of Moroccan forest fuels. J Combust.

[CR17] Hernando C, Guijarro M, Santos JA (1995). Determinacion de la relacion superficie/volumen de las aciculas muertas. Investig Agrar Sist Recur Forest.

[CR18] Hiziroglu S (2007) Dimensional changes in wood. Oklahoma Coop. Ext. Serv. NREM-5009. Division of Agricultural Sciences and Natural Resources, Oklahoma State University, pp. 1–4

[CR19] Jimenez MS, Zellnig G, Stabentheiner E, Peters J, Morales D, Grill D (2000). Structure and ultrastructure of *Pinus canariensis* needles. Flora.

[CR21] Liodakis S, Kakardakis T, Tzortzakou S, Tsapara V (2008). How to measure the particle ignitability of forest species by TG and LOI. Thermochim Acta.

[CR22] López L, Zehavi R, Climent A, Gil J (2007). Contrasting ecotypic differentiation for growth and survival in *Pinus canariensis* (*Pinaceae*). Aust J Bot.

[CR23] Martínez JP, Silva H, Ledent JF, Pinto M (2007). Effect of drought stress on the osmotic adjustment, cell wall elasticity and cell volume of six cultivars of common beans (*Phaseolus vulgaris* L.). Eur J Agron.

[CR24] Nepveu G, Jodin P (1994). Variabilité. Le bois matériau d’ingénierie.

[CR25] Papió C, Trabaud L (1990). Structural characteristics of fuel components of five Mediterranean shrubs. For Ecol Manag.

[CR26] Pardos M, Calama R, Climent J (2009). Difference in cuticular transpiration and sclerophylly in juvenile and adult pine needles relates to the species-specific rates of development. Trees.

[CR27] Pausas JG, Alessio GA, Moreira B, Corcobado G (2012). Fires enhance flammability in *Ulex parviflorus*. New Phytol.

[CR28] Pellizzaro G, Duce P, Ventura A, Zara P (2007). Seasonal variations of live moisture content and ignitability in shrubs of the Mediterranean basin. Int J Wildland Fire.

[CR29] Pereira JMC, Sequeira NMC, Carreiras JMB (1995). Structural properties and dimensional relations of some Mediterranean shrub fuels. Int J Wildland Fire.

[CR30] Piñol J, Filella I, Ogaya R, Peñuelas J (1998). Ground-based spectroradiometric estimation of live fine fuel moisture of Mediterranean plants. Agric For Meteorol.

[CR31] Rothermel RC (1983) How to predict the spread and intensity of forest and range fires. USDA Forest Service Intermountain Forest Range Experiment Station. Research Paper. General Technical Report. INT-143, 161 pp

[CR32] Valette JC (1990) Inflammabilités des espèces forestières méditerranéennes. Conséquences sur la combustibilité des formations forestières. Revue Forestière Française XLII – no. sp. 1990

[CR33] White RH, Zipperer WC (2010). Testing and classification of individual plants for fire behaviour: plant selection for the wildland–urban interface. Int J Wildland Fire.

[CR34] Zellnig G, Peters J, Jimenez MS, Morales D, Grill D, Perktold A (2002). Three-dimensional reconstruction of the stomatal complex in *Pinus canariensis* needles using serial sections. Plant Biol.

